# Diagnostic Performance of Cyclophilin A in Cardiac Surgery-Associated Acute Kidney Injury

**DOI:** 10.3390/jcm9010108

**Published:** 2019-12-31

**Authors:** Cheng-Chia Lee, Chih-Hsiang Chang, Ya-Lien Cheng, George Kuo, Shao-Wei Chen, Yi-Jung Li, Yi-Ting Chen, Ya-Chung Tian

**Affiliations:** 1 Kidney Research Center, Department of Nephrology, Chang Gung Memorial Hospital, College of Medicine, Chang Gung University, Taoyuan 333, Taiwan; chia7181@gmail.com (C.-C.L.); franwisandsun@gmail.com (C.-H.C.); yolien0205@gmail.com (Y.-L.C.); b92401107@gmail.com (G.K.); r5259@cgmh.org.tw (Y.-J.L.); 2 Graduate Institute of Clinical Medical Sciences, College of Medicine, Chang Gung University, Taoyuan 333, Taiwan; josephchen0939@gmail.com; 3 Department of Cardiothoracic and Vascular Surgery, Chang Gung Memorial Hospital, Linkou branch, College of Medicine, Chang Gung University, Taoyuan 333, Taiwan; 4 Department of Biomedical Sciences, College of Medicine, Chang Gung University, Taoyuan 333, Taiwan; ytchen@mail.cgu.edu.tw

**Keywords:** cardiovascular surgical intensive care units, cardiac surgery, acute kidney injury, cyclophilin A, neutrophil gelatinase-associated lipocalin

## Abstract

Acute kidney injury (AKI) is associated with increased morbidity and mortality and is frequently encountered in cardiovascular surgical intensive care units (CVS-ICU). In this study, we aimed at investigating the utility of cyclophilin A (CypA) for the early detection of postoperative AKI in patients undergoing cardiac surgery. This was a prospective observational study conducted in a CVS-ICU of a tertiary care university hospital. All prospective clinical and laboratory data were evaluated as predictors of AKI. Serum and urine CypA, as well as urine neutrophil gelatinase-associated lipocalin (uNGAL), were examined within 6 h after cardiac surgery. The discriminative power for the prediction of AKI was evaluated using the area under the receiver operator characteristic curve (AUROC). We found that both serum CypA and urine CypA were significantly higher in the AKI group than in the non-AKI group. For discriminating AKI and dialysis-requiring AKI, serum CypA demonstrated acceptable AUROC values (0.689 and 0.738, respectively). The discrimination ability of urine CypA for predicting AKI was modest, but it was acceptable for predicting dialysis-requiring AKI (AUROC = 0.762). uNGAL best predicted the development of AKI, but its sensitivity was not good. A combination of serum CypA and uNGAL enhanced the overall performance for predicting the future development of AKI and dialysis-requiring AKI. Our results suggest that CypA is suitable as a biomarker for the early detection of postoperative AKI in CVS–ICU. However, it has better discriminating ability when combined with uNGAL for predicting AKI in CVS-ICU patients.

## 1. Introduction

Acute kidney injury (AKI) is a severe complication after cardiac surgery and significantly affects morbidity and mortality [1,2]. Up to 15–40% of patients undergoing cardiac surgery develop AKI, with 1–6% requiring renal replacement therapy (RRT) [1,2,3,4]. The mortality rate in cardiac surgery patients with a severe, RRT-requiring AKI can be as high as 60% [3,4]. Even minor increases in serum creatinine (SCr) levels (that is, 20–25% from preoperative baseline) following cardiac surgery are associated with increased mortality [5,6]. AKI is associated with not only postoperative mortality but long-term complications, such as increased risks of myocardial infarction, heart failure, mediastinitis, and stroke [2,7,8,9]. Therefore, novel biomarkers that can predict the development and severity of AKI earlier after cardiac surgery are important tools in clinical practice.

Recently, a secreted molecule, cyclophilin A (CypA), was found to have a physiological and pathological role in cardiovascular diseases, including atherosclerosis, acute coronary syndrome, and aortic aneurysm [10,11,12,13,14]. Extracellular CypA has been found to promote either the development of atherosclerosis or the vulnerability of atherosclerotic plaques by enhancing vascular oxidative stress and inflammation [14]. CypA has also been shown to be a damage-associated molecular pattern molecule that can initiate and perpetuate the inflammatory response [15]. It can stimulate inflammatory cell recruitment and subsequent tissue injury through binding to membrane receptor CD147 [16]. Critically, Dear et al., by using a mouse model of sepsis based on cecal ligation and puncture, found that inhibition of CypA receptor CD147 attenuates sepsis-induced acute renal failure [17]. Furthermore, the increased secretion of CypA from human proximal tubular cells has been demonstrated after exposure to harmful insults, such as free radical treatment [18]. Thus, it is conceivable that urine CypA might be a potential early marker of kidney injury.

Our hypothesis was that serum CypA can be a crucial mediator leading to adverse outcomes in patients after cardiac surgery. Therefore, this study aimed to evaluate whether serum or urine CypA could be a potential marker to predict AKI after cardiac surgery.

## 2. Materials and Methods

### 2.1. Study Design

We conducted a prospective, observational study in the CVS-ICU at a tertiary care referral center in Taiwan between September 2015 and December 2016. Patients who received cardiac surgery were enrolled in this investigation. A total of 186 patients were included and divided into the AKI and non-AKI groups. Patients who had an estimated glomerular filtration rate (eGFR) < 30 mL/min/1.73 m^2^, were receiving dialysis, were aged < 18 years, or reported any prior organ transplantation were excluded. To ensure early detection, only those who underwent cardiac surgery and were admitted to the CVS-ICU within 72 h were enrolled. Demographic data, clinical characteristics, and echocardiographic data were collected. Routine biochemistry test results, such as white blood cell, hemoglobin, creatinine (Cr), and alanine aminotransferase levels were measured by the central laboratory of Chang Gung Memorial Hospital. Based on the Kidney Disease Improving Global Outcomes (KDIGO) Clinical Practice Guidelines for Acute Kidney Injury, AKI was defined under either of the following criteria: increase in SCr by ≥0.3mg/dL within 48 h or increase in SCr to ≥1.5 times the baseline within 7 days. In addition, the severity of AKI was staged according to the KDIGO guidelines [19]. The study protocol was approved by the local Institutional Review Board (number 103-1993B).

### 2.2. Clinical Assessment

All the patients received standard medical therapy after cardiac surgery. The cardiac surgical details included coronary artery bypass grafting (CABG), valve surgery, and aortic surgery. Surgical risk was assessed using the European System for Cardiac Operative Risk Evaluation (EuroSCORE) II score [20]. To determine the predictive value of potential biomarkers for AKI, the primary outcome was the development of AKI within 7 days after cardiac surgery. To assess the prognostic utility of potential biomarkers, new-onset dialysis-requiring AKI and 90-day mortality were considered secondary outcomes. After hospital discharge, a 6-month follow-up examination was performed by reviewing the electronic medical records or using telephone interviews as needed.

### 2.3. Sampling and Quantifying Urine Neutrophil Gelatinase-Associated Lipocalin (uNGAL) and Cyclophilin A (CypA)

Urine samples were collected in sterile non-heparinized tubes immediately after cardiac surgery and then centrifuged at 5000× *g* for 30 min at 4 °C to remove cells and debris. The clarified supernatants were stored at −80 °C until analysis. CypA and uNGAL were measured by an enzyme-linked immunosorbent assay using kits purchased from Cusabio Biotech (Carlsbad, CA, USA) and R&D Systems (DLCN20; Minneapolis, MN, USA), respectively, according to the manufacturers’ specifications.

### 2.4. Statistical Analysis

Continuous data, such as preoperative laboratory value, were expressed as means ± standard deviations. Since most biomarkers did not fit a normal distribution, we expressed them as median and interquartile range. Data of continuous variables for the AKI and non-AKI groups were compared using the Student’s *t*-test or Mann–Whitney U test. Fisher’s exact test was used to compare the categorical variables. The trends of uNGAL/Cr and serum CypA across chronic kidney disease (CKD) stages was assessed by the Jonckheere–Terpstra trend test. Pairwise comparisons among the CKD stages were made by the Kruskal–Wallis test with Bonferroni adjustment. The discrimination abilities of several markers (i.e., serum CypA, uNGAL/Cr, serum CypA + uNGAL/Cr, and urine CypA/Cr) in diagnosing outcomes (including AKI, hemodialysis, and 90-day mortality) were assessed using the area under the receiver operating characteristic curve (AUROC). Subsequently, optimal cut-off points and the corresponding sensitivities/specificities were obtained according to the Youden index. The areas under the curve (AUCs) of different markers were compared by the DeLong test. All tests were two-tailed, and *p* < 0.05 was considered statistically significant. No adjustment for multiple testing (multiplicity) was made in this study. Data analyses were conducted using SPSS 22 (IBM SPSS Inc, Chicago, IL, USA).

## 3. Results

### 3.1. Study Population Characteristics

Overall, 186 adult patients (116 men and 70 women) with a mean age of 60 years were investigated. AKI was diagnosed in 92 (49.5%) patients. The patient characteristics, including age, sex, preoperative laboratory data, and surgical details, are listed in Table 1. Diabetes mellitus and congestive heart failure were recorded in 32.8% and 19.9% of the patients, respectively, during recruitment. The AKI patients exhibited significantly higher EuroSCORE II than the non-AKI patients (*p* = 0.018). Furthermore, the AKI patients exhibited lower platelet and albumin levels and higher Cr levels at baseline than the non-AKI patients (*p* < 0.05; Table 1). No significant differences were seen in other clinical and biochemical parameters between the AKI and non-AKI groups after the cardiac surgeries.

Table 2 summarizes the postoperative biomarkers and clinical outcomes of the patients with and without AKI in this study. In the AKI and non-AKI groups, the median serum CypA levels were 5.8 ng/mL and 4.0 ng/mL (*p* < 0.001), respectively; the median uNGAL levels were 91 ng/mL and 31 ng/mL (*p* < 0.001), respectively; and the median urine CypA levels were 0.24 ng/mL and 0.17 ng/mL (*p* = 0.035), respectively. To compensate for perioperative variation in urine dilution, urine CypA and uNGAL were adjusted according to urine Cr. The median urine CypA/Cr levels in the AKI and non-AKI groups were 0.004 ng/mL and 0.002 ng/mL (*p* = 0.003), respectively, and the median uNGAL/Cr levels in the two groups were 1.73 ng/mL and 0.43 ng/mL (*p* < 0.001), respectively. Eventually, eleven (12%) of the AKI patients underwent hemodialysis. There were seven (7.6%) and five (5.4%) patients in the AKI group suffered from post-operative bleeding and sepsis respectively. Overall, 12 (6.5%) patients died within 90 days. Patients in the AKI group had a longer hospital stay and a higher incidence of postoperative bleeding and mortality than did those in the non-AKI group.

**Figure 1:**
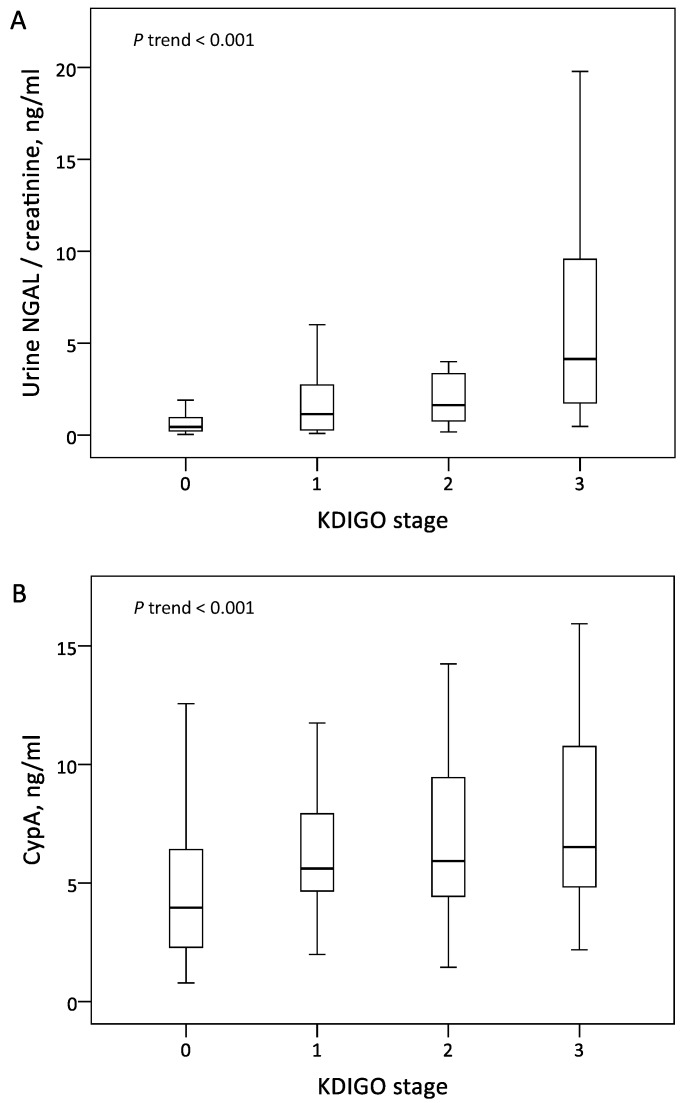
Levels of urine NGAL normalized by urine creatinine (**A**) and serum CypA (**B**) across KDIGO stages. Abbreviations: CypA, cyclophilin A; NGAL, neutrophil gelatinase-associated lipocalin; KDIGO, Kidney Disease Improving Global Outcomes.

The patients in the AKI group exhibited significantly higher serum CypA and normalized uNGAL levels than those in the non-AKI group. The level of normalized uNGAL increased along with the more severe AKI stage, but there was no significant difference between the KDIGO 1 and KDIGO 2 stages (Figure 1A). The level of serum CypA was significantly different between the AKI and non-AKI groups, but there was no significant difference among the KDIGO stages 1–3 (Figure 1B).

### 3.2. Discrimination Abilities of Serum CypA and Normalized uNGAL in Detecting AKI, Dialysis-Requiring AKI, and 90-Day Mortality

**Figure 2:**
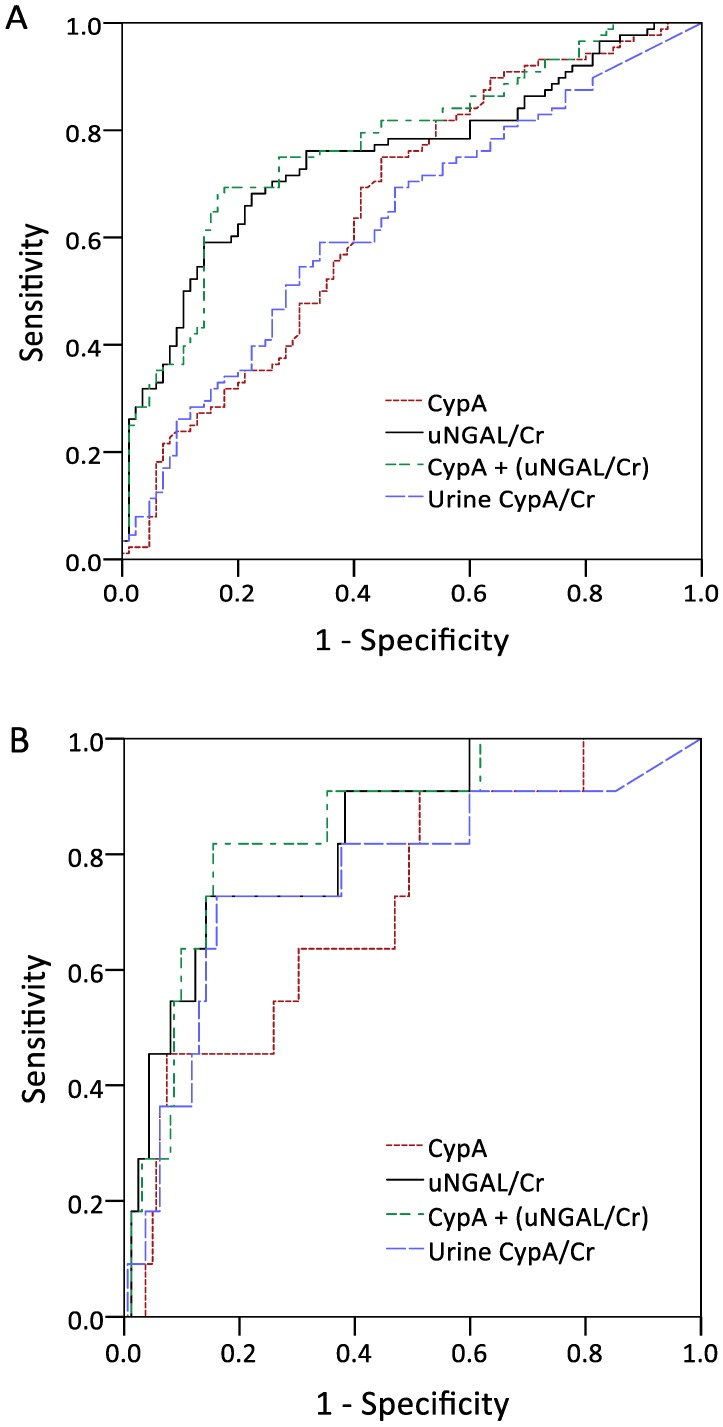

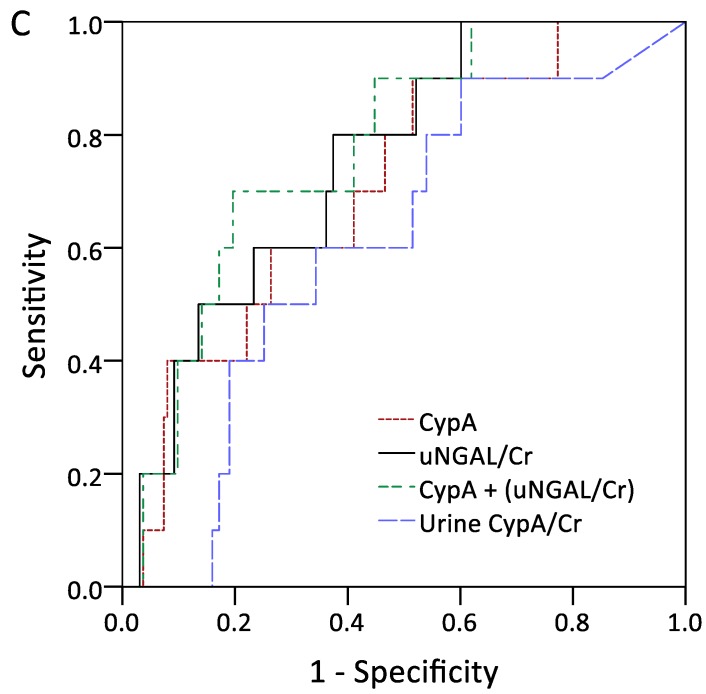
Area under the curves for serum CypA, urine NGAL normalized by urine Cr, serum CypA plus urine NGAL normalized by urine Cr, and urine CypA normalized by urine Cr in discriminating acute kidney injury (**A**), dialysis-requiring acute kidney injury (**B**), and 90-day mortality (**C**). Abbreviations: CypA, cyclophilin A; NGAL, neutrophil gelatinase-associated lipocalin; Cr, creatinine.

The performances of serum CypA and normalized uNGAL in the detection of outcomes were assessed through AUROC analysis, as shown in Figure 2A–C. The ROC analysis of serum CypA and normalized uNGAL revealed AUROC values of 0.689 (95% confidence interval [CI], 0.618–0.755) and 0.752 (95% CI, 0.684–0.812), respectively, for predicting the future development of AKI. The combination of serum CypA and normalized uNGAL showed the highest AUC of 0.787 (95% CI, 0.721–0.843) in diagnosing AKI. In terms of using a single marker to predict dialysis-requiring AKI, serum CypA and normalized uNGAL had AUROCs of 0.738 (95% CI, 0.668–0.799) and 0.835 (95% CI, 0.773–0.885), respectively. Normalized urine CypA had an AUROC of 0.762 and exhibited a good specificity of 83.9% for a cutoff value of 0.012. The combination of CypA and normalized uNGAL showed the highest AUC of 0.848 (95% CI, 0.788–0.896).

As for the detection of AKI, CypA exhibited a sensitivity of 76.1% and a specificity of 58.5% for a threshold value of 4.36 ng/mL, whereas normalized uNGAL exhibited a poor sensitivity of 68.5% and a specificity of 76.6% for a cut-off value of 0.85 (Table 3). However, there was no significant difference in the 90-day survival rates between the subgroups of high/low serum CypA and normalized uNGAL.

## 4. Discussion

The development of AKI is associated with unfavorable outcomes and high mortality in patients undergoing cardiac surgery. Because renal dysfunction is known as a well-established predictor of all-cause mortality in cardiac surgery, biomarkers for the early detection of AKI after cardiac surgery would be valuable for clinical practices, such as decision making, patient counseling, and optimization of post-operative care [21]. NGAL has been the most popular biomarker for the early identification of AKI following cardiac surgery. However, compared with the excellent discrimination reported in pediatric patients, studies of urinary NGAL in adult patients reported only moderate discrimination, with an AUROC of 0.72 (95% confidence interval, 0.66–0.79) [22]. Thus, new biomarkers with better performance are urgently needed. Strategies combining biomarkers with different types of pathophysiological relevance may also be beneficial in risk stratification. In this study, we found that both serum CypA and normalized urine CypA were elevated in the patients who developed AKI after sample collection. As we have shown, serum CypA is suitable for the early detection of AKI in patients undergoing cardiac surgery, with a good sensitivity and acceptable discriminative power comparable to those of normalized uNGAL. A combination of serum CypA and normalized uNGAL enhanced the overall performance for predicting the development of AKI and dialysis-requiring AKI, with AUROC values of 0.787 and 0.848, respectively.

Cyclophilins are a family of ubiquitous proteins that are evolutionarily well conserved and present in all prokaryotes and eukaryotes [23]. The most abundant member of this family is CypA, which accounts for about 0.1–0.6% of total cytosolic proteins [24]. Although CypA is present intracellularly and was originally identified as the primary cytosolic binding protein of the immunosuppressive drug cyclosporin A, it has been found to be secreted from cells in response to inflammatory stimuli, such as hypoxia, infection, and oxidative stress [25,26,27,28]. Secreted CypA has been demonstrated to be a damage-associated molecular pattern molecule that has a potent chemotactic effect on leukocytes, and, in turn, perpetuates the inflammatory response [15,27]. Moreover, high levels of extracellular CypA have also been detected in several different human inflammatory diseases, such as rheumatoid arthritis [29,30] and sepsis [17,31], and found to be correlated with the severity of those diseases. Our data are in accordance with these findings. The distinctive characteristics of cardiac surgery, including aortic clamping and cardiopulmonary bypass, which induce a systemic inflammatory response lead to the development of AKI [32]. Notably, our study also revealed that urine CypA is elevated in patients with AKI. Because CypA is an 18-kDa protein that theoretically can be freely filtered by renal glomeruli, higher levels of urine CypA in patients with AKI may just reflect an increase in serum CypA levels. An alternative explanation is that the actual source of urine CypA may be the injured renal tubular cells. Studies have demonstrated that CypA is highly expressed in the kidney, especially in the proximal tubular epithelial cells [33]. Tsai et al. also demonstrated that CypA is released by human proximal tubular cells in a dose-dependent manner after exposure to free radical treatment [18]. However, our study showed that normalized urine CypA only exhibited modest discrimination ability for predicting AKI, with an AUROC of 0.63. In addition, although normalized urine CypA exhibited a good specificity of 83.9% in predicting dialysis-requiring AKI for a cutoff value of 0.012, its overall performance was not better than that of the well-known marker normalized urine NGAL. Intriguingly, previous studies reported that not only urine soluble components, but human urine exosomes, contain CypA [15]. Whether the exosomal part of urine CypA can exhibit better discrimination power than soluble part of urine CypA alone in stratifying AKI risk needs further investigation.

Our study found that serum CypA levels were higher in the patients who subsequently developed AKI than in those who did not, but the levels were not significantly different among the AKI KDIGO stages 1–3. This might be explained by the notion that the AKI severity is more associated with changes in the serum CypA level over time than with the level at a single time. Indeed, some of the elevation in serum CypA levels in our patients might just reflect their underlying cardiac diseases. Extracellular CypA was previously found to contribute to cardiovascular diseases as a novel player not only through its proinflammatory actions but through its proatherogenic properties [10,11,34]. Yan et al. reported that serum CypA concentrations in patients with unstable angina and acute myocardial infarction were significantly higher than those in patients with stable angina and controls [12]. Serum CypA levels were also previously found to be associated with the clinical outcomes of coronary artery disease [35], ST-elevated myocardial infarction [36], and heart failure [37]. In addition to coronary artery disease, extracellular CypA was identified as a mediator in abdominal aortic aneurysm (AAA) progression. In human AAA lesions, CypA was highly expressed, especially in the area that expresses active metalloproteinase 2 (MMP-2) [14]. Using human AAA-derived vascular smooth muscle cells, angiotensin II induces the release of CypA and enhances vascular inflammation by activating MMP activity, which was significantly reduced by treatment with the CypA inhibitor. Based on these evidences, we hypothesized that increased serum CypA levels may partly be a consequence of underlying cardiovascular diseases, and that patients with more severe cardiovascular diseases might have hemodynamic instability, leading to higher baseline renal dysfunction. Further studies using preoperative serum CypA levels or serum CypA dynamics are needed to help clarify the relationship between CypA and the outcomes of cardiac surgery.

Our study showed that normalized uNGAL best predicted the development of AKI, and the level of normalized uNGAL increased along with the more severe AKI stage. This finding is consistent with previous reports that uNGAL is an early predictive biomarker of AKI following cardiac surgery [38,39]. NGAL was originally identified from neutrophils as a shuttle for iron transport, and its upregulation has been demonstrated in the proximal renal tubule after exposure to harmful insults, such as ischemia [40]. Consequently, increased level of NGAL is rapidly detectable in the urine before SCr is elevated, presumably resulting from acute tubular damage. Although the discrimination ability of CypA levels regarding the development of AKI was not superior to that of normalized uNGAL levels, our study found that normalized uNGAL had only modest sensitivity in predicting AKI and dialysis-requiring AKI. Meanwhile, we further found that the combination of these two biomarkers provides the most accurate predictive ability in these patients, suggesting that their combination is a reasonable strategy to improve the diagnostic performance of biomarkers. Our study also found a moderate discrimination ability of normalized urine CypA when predicting dialysis-requiring AKI, with comparable sensitivity and specificity ability when compared to the well-known marker normalized uNGAL. However, this study only considered AKI identified within a 7-day period; thus, studies focusing on longer-term outcomes, such as acute kidney disease [41], are warranted to clarify the potential pathogenic role of serum CypA or urine CypA in cardiac surgery-related kidney injury.

This study has several limitations. First, only one measurement of the CypA and NGAL levels was used in this cross-sectional study to predict the development of AKI. Repeated measurements to detect persistent or secondary kidney damage may improve the predictive ability. Second, the roles and expression of CypA in AKI require further investigation. Using animal models to evaluate the origin of CypA in urine may help determine the pathogenic role of urine CypA. Third, this research was conducted on a heterogeneous population with different cardiovascular diseases, and no subgroup analysis was conducted to explore the relationships between a specific disease type and the biomarkers. We also did not analyze the relationships between CypA levels and medications that may interfere with CypA levels. Finally, given the small sample size and observational design, additional prospective trials are warranted to explore the role of CypA in different etiologies of AKI.

In summary, both serum CypA and normalized uNGAL are suitable for the early detection of AKI in patients undergoing cardiac surgery, as both have acceptable discriminative power. Moreover, the combination of these two markers provides the highest AUROC and could serve as a new non-invasive test for use in clinical applications to differentiate AKI and RRT, potentially shortening the time to the initiation of appropriate therapy. Relatedly, the careful consideration of the appropriate medication, choice of therapy, and early intervention in patients exhibiting increased biomarker levels may improve AKI outcomes.

## Figures and Tables

**Table 1:** Baseline characteristics of the patients with and without AKI after cardiac surgeries.

Characteristics	All Patients	AKI	Non-AKI	*p*
Patient number	186	92	94	-
Age, year	60.0 ± 14.6	60.7 ± 14.8	59.3 ± 14.5	0.504
Male gender, *n* (%)	116 (62.4)	55 (59.8)	61 (64.9)	0.545
Diabetes mellitus, *n* (%)	61 (32.8)	33 (35.9)	28 (29.8)	0.436
CHF NYHA III/IV, *n* (%)	37 (19.9)	22 (23.9)	15 (16.0)	0.201
Mean arterial pressure, mmHg	90.3 ± 14.4	89.5 ± 15.4	91.0 ± 13.3	0.475
LVEF, %	60.9 ± 15.5	59.9 ± 15.9	61.8 ± 15.3	0.394
Preoperative laboratory data				
Leukocyte count, 1000/mL	7.8 ± 3.4	7.7 ± 3.6	7.9 ± 3.2	0.730
Hemoglobin, g/dL	12.6 ± 2.4	12.3 ± 2.7	12.9 ± 2.0	0.083
Platelet count, 1000/mL	201 ± 75	189 ± 81	212 ± 66	0.038
ALT, u/L	30.2 ± 34.8	31.2 ± 43.3	29.1 ± 24.0	0.691
Serum creatinine, mg/dL	1.1 ± 1.0	1.3 ± 1.3	0.9 ± 0.4	0.013
Albumin, mg/dL	3.9 ± 0.5	3.9 ± 0.6	4.0 ± 0.4	0.044
EuroSCORE II	6.7 (6.1)	8.0 (7.2)	5.5 (4.5)	0.018
Surgical detail, *n* (%)				0.162
CABG	61 (32.8)	24 (26.1)	37 (39.4)	
Valve surgery	64 (34.4)	33 (35.9)	31 (33.0)	
CABG + valve surgery	17 (9.1)	12 (13.0)	5 (5.3)	
Aorta	34 (18.3)	19 (20.7)	15 (16.0)	
Others	10 (5.4)	4 (4.3)	6 (6.4)	

Continuous data are presented as means ± SDs or medians (interquartile range); AKI, acute kidney injury; CHF, congestive heart failure; NYHA, New York Heart Association; ALT, alanine aminotransferase; CABG, coronary artery bypass grafting.

**Table 2:** Postoperative biomarkers and outcomes of the patients with and without AKI after cardiac surgeries.

Characteristics	All Patients	AKI	Non-AKI	*p*
Patient number	186	92	94	-
Postoperative biomarkers				
Urine NGAL, ng/mL	44 (104)	91 (141)	31 (39)	<0.001
Urine NGAL/urine creatinine	0.73 (1.9)	1.73 (6.51)	0.43 (0.65)	<0.001
CypA, ng/mL	5.2 (3.3)	5.8 (3.9)	4.0 (4.3)	<0.001
Urine CypA, ng/mL	0.19 (0.29)	0.24 (0.40)	0.17 (0.24)	0.035
Urine CypA/urine creatinine	0.003 (0.007)	0.004 (0.013)	0.002 (0.005)	0.003
Peak serum creatinine, mg/dL	1.6 ± 1.3	2.3 ± 1.8	1.0 ± 0.4	<0.001
Outcome				
AKI stage 1/2/3	-	48/23/21	-	-
Renal replacement therapy, *n* (%)	12 (6.5)	11 (12.0)	1 (1.1)	0.002
Postoperative bleeding, *n* (%)	8 (4.3)	7 (7.6)	1 (1.1)	0.034
Postoperative sepsis, *n* (%)	6 (3.2)	5 (5.4)	1 (1.1)	0.116
Stay of hospital, days	21.4 (15.0)	28.0 (18.5)	14.9 (11.0)	<0.001
Mortality in 90 days, *n* (%)	12 (6.5)	10 (10.9)	2 (2.1)	0.018

Continuous data are presented as means ± SDs or medians (interquartile range); AKI, acute kidney injury; NGAL, neutrophil gelatinase-associated lipocalin; CypA, cyclophilin A.

**Table 3:** Diagnostic property of markers in discriminating outcomes.

Outcome/Marker	AUC (95% CI)	Cut-Off #	Sensitivity (95% CI)	Specificity (95% CI)
Acute kidney injury				
CypA	68.9 (61.8–75.5)	>4.36	76.1 (66.1–84.4)	58.5 (47.9–68.6)
Urine NGAL/Cr	75.2 (68.4–81.2)	>0.85	68.5 (58.0–77.8)	76.6 (66.7–84.7)
CypA + (urine NGAL/Cr)	78.7 (72.1–84.3)	NA	NA	NA
Urine CypA/Cr	63.0 (55.3–70.2)	>0.003	59.1 (48.1–69.5)	65.9 (54.8–75.8)
Dialysis-requiring AKI				
CypA	73.8 (66.8–79.9)	>4.84	91.7 (61.5–99.8)	50.0 (42.3–57.7)
Urine NGAL/Cr	83.5 (77.3–88.5)	>3.09	75.0 (42.8–94.5)	85.1 (78.9–90.0)
CypA + (urine NGAL/Cr)	84.8 (78.8–89.6)	NA	NA	NA
Urine CypA/Cr	76.2 (69.2–82.3)	>0.012	72.7 (39.0–94.0)	83.9 (77.4–89.2)
90-day mortality				
CypA	67.0 (59.7–73.7)	>4.84	83.3 (51.6–97.9)	49.4 (41.8–57.1)
Urine NGAL/Cr	75.4 (68.5–81.4)	>1.12	83.3 (51.6–97.9)	62.6 (55.0–69.8)
CypA + (urine NGAL/Cr)	73.1 (66.2–79.4)	NA	NA	NA
Urine CypA/Cr	61.1 (53.4–68.4)	>0.0016	90.0 (55.5–99.7)	39.9 (32.3–47.8)

AUC, area under the curve; CI, confidence interval; CypA, cyclophilin A; NGAL, neutrophil gelatinase-associated lipocalin; Cr, creatinine; AKI, acute kidney injury; NA, not applicable; #, number by Youden index.
